# Pharmacogenetics in pediatric cardiology

**DOI:** 10.3389/fped.2026.1911224

**Published:** 2026-09-21

**Authors:** Adenike T. Adenikinju, Jonathan B. Wagner, Mollie M. Walton

**Affiliations:** 1 Department of Pediatrics, University of Missouri-Kansas City School of Medicine, Kansas, MO, United States; 2 Ward Family Heart Center, Children’s Mercy Kansas City, Kansas, MO, United States; 3 Division of Clinical Pharmacology, Toxicology and Therapeutic Innovation, Children’s Mercy, Kansas, MO, United States

**Keywords:** adverse drug events, guidelines, pediatric cardiology, pharmacogenetics, precision medicine

## Abstract

Children with congenital and acquired heart disease frequently require complex and prolonged pharmacotherapy, often involving off-label cardiovascular medications with narrow therapeutic ranges. Consequently, variability in drug response and medication-related adverse events remains a significant clinical challenge in pediatric cardiology. Pharmacogenetics provides a framework to individualize drug selection and dosing by incorporating genetic variability alongside established clinical and developmental factors. This review outlines the current clinical utility and emerging role of pharmacogenetics in pediatric cardiology, emphasizing applications most relevant to day-to-day patient care. Established pharmacogenetic associations with direct implications for pediatric cardiac practice include gene–drug pairs involving tacrolimus (*CYP3A5*), warfarin (*CYP2C9*, *VKORC1, CYP4F2,* rs12777823 within the *CYP2C)*, clopidogrel (*CYP2C19)*, flecainide (*CYP2D6)*, metoprolol (*CYP2D6)*, and statins (*SLCO1B1, ABCG2, CYP2C9*), many of which are supported by guideline-based recommendations from the Clinical Pharmacogenetics Implementation Consortium. This review also highlights pharmacogenetic considerations for commonly prescribed non-cardiac medications that may influence cardiovascular outcomes in children with heart disease, identifies emerging areas for research, and emphasizes that as pharmacogenetic testing becomes increasingly accessible, its thoughtful integration into pediatric cardiology practice may improve therapeutic precision, enhance safety, and reduce preventable adverse drug events.

## Introduction

With emerging techniques that can deliver precision therapeutics to the individual patient, pediatric cardiac patients may stand to benefit the most from adoption of pharmacogenetic guidance, given the specialized drug therapy required in the management of this unique population. Although strides have been made in pediatric drug labeling (1), labeling for pediatric cardiovascular therapeutics remains significantly inadequate (2). Alarmingly, at least 80% of pediatric cardiac patients receive at least one off-label medication and nearly 30% receive three or more (3–5). As a result, insufficient therapeutic response and adverse events rates are common among children, adolescents, and young adults with congenital and acquired heart disease, emphasizing the urgent need for prospective evaluation of drug safety and effectiveness in this population (6–8).

Traditional drug development and optimization rely on broad, largely homogenous cohorts and, subsequently, extrapolate the data to individual patients. In pediatric cardiology, this problem is amplified, as evidence is almost exclusively from adults with acquired cardiovascular disease, as opposed to congenital heart disease (CHD), and is then applied to children of varying ages, developmental stages, and underlying pathophysiology. However, strides to address the knowledge gaps in the developmental trajectories of drug disposition, namely drug metabolism (9–12) and transporters (13) have been accomplished. Unfortunately, data on the impact of genetic variation on drug disposition and response in pediatric cardiovascular patients is limited.

Before precision-based care strategies, such as pharmacogenetic-guided dosing, can be effectively implemented, pediatric cardiac providers must first appreciate both pharmacologic properties of the administered drug and the unique patient factors that could potentially influence the dose-exposure-response relationship for that individual pediatric cardiac patient. Many of the factors have previously been described in several comprehensive reviews (14–19). Importantly, sources of variability in drug disposition (i.e., the dose-exposure relationship) should be defined before linking differences in drug response to genetic variation in drug targets. Genetic variation in the protein(s) responsible for drug absorption, distribution, metabolism, and excretion is illustrated in Figure 1. Developmental ontogeny, which describes age-related changes in drug-metabolizing enzymes and transporters, further complicates matters (19). Although additional prospectively-generated data in the pediatric cardiac patient is urgently needed, there is emerging evidence to support pharmacogenetic-informed treatment guidelines, which will be discussed herein.

**Figure 1:**
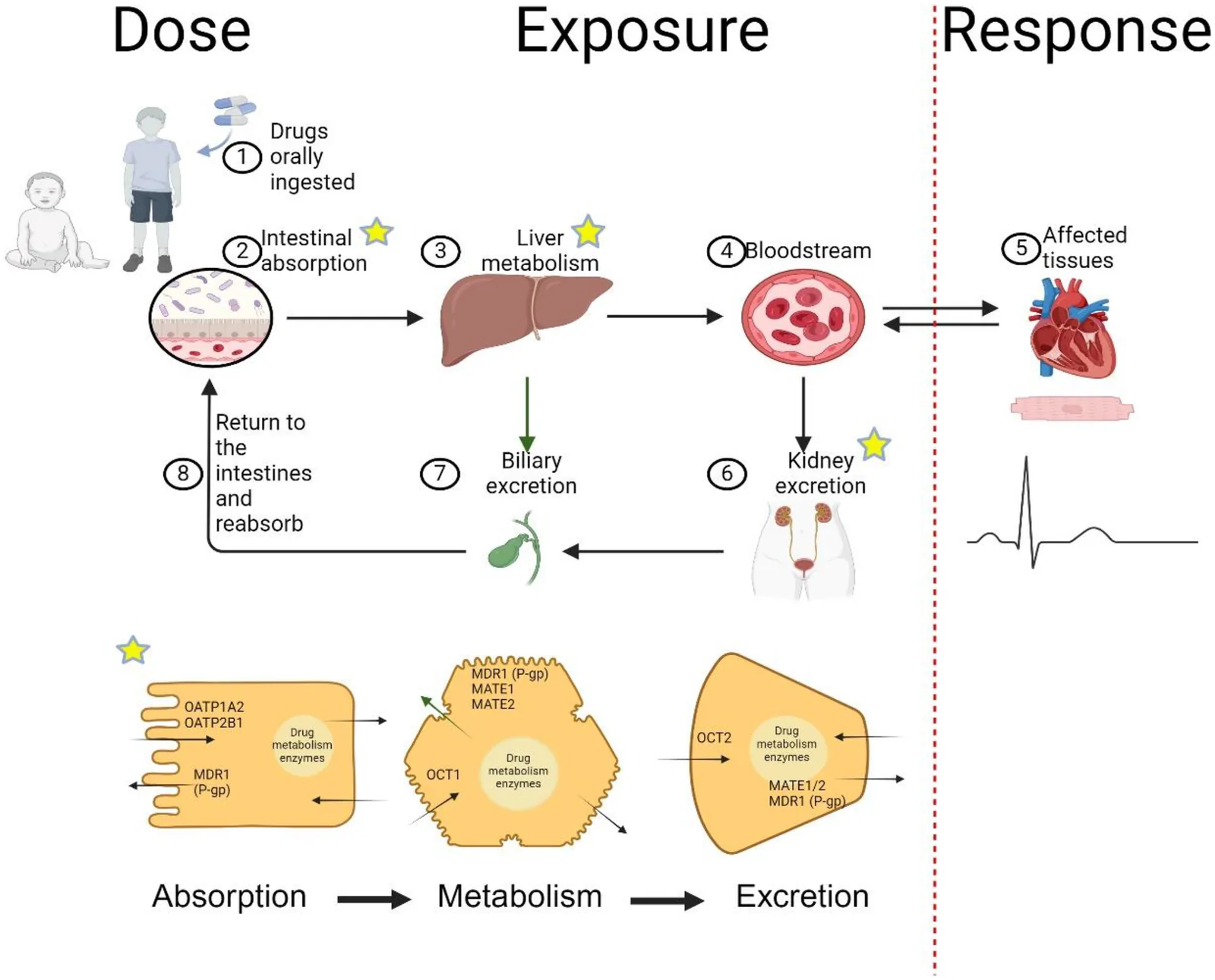
Dose-Exposure-Response relationship. Reproduced with permission from:Walton, M. and J.B. Wagner, Pediatric Beta Blocker Therapy: A Comprehensive Review of Development and Genetic Variation to Guide Precision-Based Therapy in Children, Adolescents, and Young Adults. Genes (Basel), 2024. 15(3).

## Current state of pharmacogenetics guidelines in pediatric cardiology

Pharmacogenetics in pediatric cardiology remains in the early stages of clinical implementation, with the strongest evidence and guideline-supported applications in cardiac transplantation, anticoagulation and antiplatelet optimization, and, more recently, antiarrhythmic therapy. The Clinical Pharmacogenetics Implementation Consortium (CPIC) facilitates pharmacogenetic testing and offers evidence-based guidelines for patient care (Table 1). While there is a paucity in evidence relating specifically to pediatric pharmacokinetics, the following is an outline of the current knowledge of drug-gene pairs, including recent updates outlined by CPIC.

**Table 1:** CPIC pharmacogenetic guidelines for select gene-drug pairs.

Medication	Primary Gene(s)	Clinical Indication	Key CPIC Recommendation(s)	CPIC Guideline (PMID)
Tacrolimus	*CYP3A5*	Immunosuppression	CYP3A5 expressers (*CYP3A5 1* carriers) require higher starting doses to achieve target trough concentrations; non-expressers require standard dosing.	25,801,146
Clopidogrel	*CYP2C19*	Antiplatelet therapy	CYP2C19 poor or intermediate metabolizers have reduced activation and efficacy; alternative antiplatelet therapy is recommended.	25,689,255
Warfarin	*CYP2C9*, *VKORC1*, *CYP4F2*	Anticoagulation	Genotype-guided initial dosing is recommended to reduce bleeding risk and improve time in therapeutic range.	28,198,005
Atomoxetine	*CYP2D6*	ADHD	CYP2D6 poor metabolizers have higher exposure and increased adverse effects; dose reduction or alternative therapy recommended.	30,801,677
Selective Serotonin Reuptake Inhibitors	*CYP2D6*, *CYP2C19* (± others)	Depression, anxiety	Genotype-guided dose adjustments or alternative selection based on metabolizer status to optimize efficacy and tolerability.	37,032,427
Omeprazole	*CYP2C19*	Acid suppression (GERD, ulcers)	CYP2C19 rapid/ultrarapid metabolizers may require higher doses or alternative PPIs; poor metabolizers may need dose reduction.	32,770,672

### Tacrolimus

Tacrolimus is an immunosuppressant with a narrow therapeutic index used after pediatric heart transplantation, whose metabolism is largely mediated by the polymorphic CYP3A5 enzyme (20), resulting in extensive, intermediate, and poor metabolizer phenotypes. CYP3A5 expression, and resultant metabolic activity, is associated with lower trough levels of tacrolimus (i.e., increased clearance rates), compared to the absence of CYP3A5 non-expression (20). This means that CY3A5 metabolizers may require higher doses of tacrolimus compared to non-metabolizers to achieve therapeutic concentrations (20). Approximately 80%-85% of individuals with European ancestry, 50% with Asian ancestry, and 30% with African ancestry are CYP3A5 non-expressors (21). Inadequate drug exposure (i.e., CYP3A5 metabolizers) is associated with increased risk for organ rejection due to lower serum levels of tacrolimus, while excessive drug exposure (i.e., CYP3A5 non-metabolizers) increases toxicity risk, including nephrotoxicity and neurotoxicity.

CPIC does not recommend for or against routine genetic testing in transplant patients, but offers recommendations based on whether genetic information is available and known (20). However, pre-emptive genotyping represents an opportunity for personalized tacrolimus therapy. A study of 45 pediatric kidney transplant patients found that CYP3A5 metabolizers required significantly higher doses during the 6-month post-transplant period, and CYP3A5 correlated with rejection rates (22).

### Warfarin

Pediatric cardiology patients use warfarin, an oral anticoagulant, for a variety of indications, such as prophylaxis in the presence of mechanical prosthetic heart valves, Fontan circulation, and Kawasaki disease associated with coronary artery abnormalities. Warfarin inhibits vitamin K epoxide reductase, impairing vitamin K-dependent activation of coagulation factors II, VII, IX, and X, as well as proteins C and S. Warfarin is a racemic mixture and CYP2C9 metabolizes the more potent S-enantiomer*.*

*CYP2C9* and *VKORC1* polymorphisms are the primary genetic determinants of warfarin pharmacokinetics and pharmacodynamics, collectively explaining approximately 21% of dose variability seen in children (23, 24). The *CYP2*C9 polymorphisms CYP2C9*2 and CYP2C9*3 result in decreased warfarin clearance and thus lower dose requirements to achieve therapeutic effect (25, 26). *VKORC1* encodes vitamin K epoxide reductase. The common promoter polymorphism in *VKORC1* refers to a single-nucleotide variant (−1639G > A), which affects the hepatic mRNA expression. VKORC1 A-allele carriers have reduced gene expression and lower warfarin dose requirements and, together with *CYP2C9* variants, *VKORC1* genotype significantly influences warfarin maintenance dose, time to therapeutic INR, and risk of over-anticoagulation in pediatric patients (24, 25, 27–29). CYP4F2 catalyzes vitamin K oxidation, and the *V433M* variant shows decreased activity, requiring higher warfarin doses to achieve anticoagulation. Additionally, the single nucleotide variant rs12777823 within the *CYP2C* gene cluster exerts a clinically relevant effect on warfarin dose in individuals of African descent (30).

The 2017 CPIC guideline update identifies the genetic variants *CYP2C9, VKORC1, CYP4F2* and rs12777823 as key determinants of warfarin dose requirements and provides genotype-guided dosing recommendations for adults and children that integrate genetic and clinical factors (30). It is important to note that maintaining therapeutic drug monitoring with INR is necessary, regardless of genotype, as non-genetic factors (e.g., diet) remain important (30).

### Clopidogrel

Clopidogrel, an oral antiplatelet agent, is used as prophylaxis in the prevention of clot formation in pediatric cardiac patients with increased risk, such as those with medical devices, including intravascular stents, mechanical valves, and pulmonary flow restrictors (31). Clopidogrel undergoes hepatic metabolism in which its active metabolite selectively and irreversibly binds to P2Y12 receptors, thus blocking adenosine diphosphate-mediated platelet aggregation. *CYP2C19* is the primary genetic determinant of clopidogrel response, with loss-of-function alleles associated with reduced active metabolite formation, diminished platelet inhibition, and thus increasing risk for major cardiovascular events in intermediate and poor metabolizers (32, 33). Worse clinical outcomes have been observed in adult patients undergoing percutaneous coronary intervention, and most studies on clopidogrel have been limited to adults with acute coronary syndrome.

The 2022 CPIC guideline update recommends avoiding standard-dose clopidogrel in both intermediate and poor metabolizers undergoing acute coronary syndrome and/or percutaneous coronary intervention, with alternative P2Y12 inhibitors (e.g., prasugrel or ticagrelor) being preferred (32, 34). While pediatric-specific data for clopidogrel pharmacogenetics are limited, there have been case reports that reinforce its clinical relevance. Five children receiving clopidogrel with intermediate or poor CYP2C19 metabolism experienced a lack of therapeutic benefit, including three pre-cardiac transplant patients who had thrombotic events (35).

### Flecainide

Flecainide is a class 1C antiarrhythmic drug with a narrow therapeutic index used in the treatment of arrhythmias, such as supraventricular tachycardia, atrial flutter and fibrillation and is metabolized mainly by the CYP2D6 enzyme. Genetic variation in *CYP2D6* affects its pharmacokinetics and subsequently its serum concentrations, making genotyping data useful due to the drug's narrow therapeutic index (36). This is particularly important in children whose drug disposition and response pathways may be developmentally immature (16). Poor and intermediate metabolizers may have decreased clearance of flecainide, leading to increased plasma concentration and higher risk of drug-toxicity related adverse effects, such widening of the QRS interval and a consequent proarrhythmic state. In contrast, ultrarapid metabolizers may process flecainide more quickly, which then may lead to reduced efficacy, although data on ultrarapid metabolizers are limited (36). While CYP2D6 becomes active within hours to days after birth, the CYP1A2 enzyme (which serves as an alternate metabolic pathway for flecainide, particularly in CYP2D6 poor or intermediate metabolizers), does not become active until 1-3 months after birth. This creates a window in early infancy where neonates with reduced CYP2D6 function cannot compensate through CYP1A2 metabolism, potentially leading to drug toxicity (16). CYP2D6 is genetically absent in approximately 7%-10% of the population, while intermediate metabolizers comprise approximately 20%-40% depending on ancestry (37, 38).

While the CPIC has not published a formal guideline specifically for flecainide, the Dutch Pharmacogenetics Working Group (DPWG) provides recommendations for each phenotypic group. This includes flecainide initiation with reduced dosing for known poor and intermediate metabolizers with corresponding plasma level monitoring and serial ECGs to direct further titration. Ultrarapid metabolizers may require higher doses to achieve therapeutic effect, and one should consider alternative antiarrhythmic agents not primarily metabolized by CYP2D6, especially in the case of lack of efficacy (39). A case report described an infant with recurrent supraventricular tachycardia who developed complete heart block requiring ECMO support after receiving a standard therapeutic dose of flecainide. The infant was subsequently found to be a CYP2D6 intermediate metabolizer with supratherapeutic flecainide serum concentrations (40).

### Metoprolol

Metoprolol is a cardio-selective beta-1 adrenergic receptor blocker used in the management of numerous cardiovascular conditions, including supraventricular tachycardia, hypertrophic cardiomyopathy, cardiac channelopathies, and aortic root dilation. Metoprolol is metabolized by CYP2D6, genetic variation in *CYP2D6* significantly influences systemic drug exposure. Poor metabolizers exhibit significantly higher systemic drug exposure and greater pharmacodynamic effects, including larger reductions in heart rate and blood pressure compared to normal metabolizers, with differences up to 13-fold between ultrarapid and poor metabolizers (41, 42). In contrast, ultrarapid metabolizers may have lower drug exposure and reduced therapeutic response.

The 2024 CPIC guideline provides recommendations for metoprolol, making it the only beta-blocker with CPIC-level evidence of genotype-guided dosing (43). For poor metabolizers, CPIC recommends initiating metoprolol at the lowest recommended dose with careful titration and monitoring for adverse effects; alternative beta-blockers not primarily metabolized by CYP2D6 may be considered if intolerance occurs. Standard dosing is recommended for normal and intermediate metabolizers, whereas current evidence is insufficient to support specific dosing recommendations for ultrarapid metabolizers. Although pediatric-specific data remain limited, *CYP2D6* genotype appears to correlate with CYP2D6 enzyme activity by about two weeks of age, supporting cautious extrapolation to the pediatric population.

### Statins

With strong evidence linking childhood subclinical atherosclerosis to adult coronary artery disease outcomes (44–48), more children in the United States now qualify for treatment aimed at addressing dyslipidemia, an important contributor to coronary artery disease (49). 3-Hydroxy-3-methyl-glutaryl Coenzyme A (HMG-CoA) reductase inhibitors, also known as statins, are the most frequently prescribed lipid-lowering medications when lifestyle changes alone are insufficient.

Although statins effectively lower low-density lipoprotein cholesterol in pediatric populations, there is substantial interindividual variability in systemic exposure and treatment response (49). Genetic variation in proteins involved in statin disposition, particularly in the hepatic uptake transporter *SLCO1B1*, can significantly alter statin pharmacokinetics (50–56). Reduced transporter activity results in increased systemic exposure, which is associated with increased risk of adverse events, with statin-associated musculoskeletal symptoms being the most common (57, 58).

The CPIC 2022 guideline provides evidence-based recommendations for tailoring statin therapy derived from individual *SLCO1B1, ABCG2, CYP2C9* genotypes to mitigate the risk of statin-associated musculoskeletal symptoms, while preserving cardiovascular benefits (59). For individuals with decreased-function *SLCO1B1* variants, CPIC recommends consideration of lower-risk statins, lower starting doses, or enhanced monitoring depending on the specific statin prescribed (59).

While pediatric-specific CPIC recommendations are not currently available, emerging evidence suggests that the genotype-phenotype relationship observed in adults may be applicable to the pediatric cohort for pravastatin rosuvastatin, and atorvastatin (53–55). However, pediatric studies of simvastatin have demonstrated pronounced effect of *SLCO1B1* genotype, with each copy of the c.521C variant associated with an approximately 2.5-fold increase in systemic exposure (56). Additional genotype-stratified pediatric studies are needed to support development of formal pharmacogenetic prescribing recommendations for children receiving statin therapy.

### Other medications

Beyond cardiac-specific drugs, atomoxetine, selective serotonin reuptake inhibitors (SSRIs), and proton pump inhibitors (PPIs)—used to treat attention deficit hyperactivity disorder (ADHD), mood disorders, and gastroesophageal reflux disease (GERD), respectively—are frequently prescribed in pediatric cardiac patients and have clinically actionable pharmacogenetic associations. Children with CHD have significantly elevated prevalence of ADHD, with rates as high as 40%-50% in some CHD populations (60). Atomoxetine, a nonstimulant medication for ADHD, exhibits significant CYP2D6-dependent variability in systemic exposure, leading to genotype-based dosing recommendations. Compared with CYP2D6 normal metabolizers, poor metabolizers have higher drug concentrations and increased risk of greater cardiovascular effects, such as increased heart rate and diastolic blood pressure, as well as higher frequency of decreased appetite (61, 62). These observations formed the basis for CPIC recommendations supporting CYP2D6-guided atomoxetine dosing, making it the only ADHD medication currently addressed by CPIC guidelines (61, 63, 64).

SSRIs, including citalopram, escitalopram, fluoxetine, fluvoxamine, paroxetine, and sertraline, undergo metabolism through CYP2D6 and/or CYP2C19 pathways, resulting in genotype-dependent differences in drug exposure and treatment response. Children with CHD have significantly elevated prevalence of anxiety and depression compared to children without CHD (60, 65) and SSRIs have important cardiovascular effects in CHD patients, such as the risk of QTc interval prolongation and arrhythmia. CPIC guidelines incorporate CYP2D6 and CYP2C19 genotype information to guide antidepressant selection and dosing in an effort to optimize efficacy and tolerability (66).

Proton pump inhibitors including omeprazole, pantoprazole, lansoprazole, esomeprazole are metabolized primarily by CYP2C19, and genetic variation in *CYP2C19* can significantly affect drug exposure and therapeutic response. This is particularly relevant because children with CHD have elevated rates of gastrointestinal comorbidities, including conditions requiring acid suppression therapy such as GERD and necrotizing enterocolitis (67, 68). CPIC guidelines provide *CYP2C19* genotype-based recommendations for proton pump inhibitor dosing and selection (69).

## Emerging pharmacogenetics in pediatric cardiology

Pulmonary hypertension (PH), a chronic, progressive condition where the pulmonary arteries become narrow, thick, or rigid, impairing blood flow, is a significant complication in pediatric cardiac patients, particularly those with CHD. Three primary drug classes are used for treatment to address endothelial dysfunction and vascular remodeling that underlies PH. These include phosphodiesterase-5 inhibitors (sildenafil, tadalafil), endothelin receptor antagonists (bosentan, ambrisentan, macitentan), and prostacyclins (epoprostinil, treprostinil, iloprost). Bosentan is the agent with the most well-characterized pharmacogenetic information, with the CYP2C9*2 variant being associated with bosentan-induced hepatotoxicity (70). Bosentan is also a substrate of the OATP1B1/1B3 hepatic transporters, which explains the contraindication with cyclosporine in post-transplant patients.

Sildenafil is metabolized primarily by the CYP3A4 enzyme, making drug-drug interactions clinically important. CYP3A4 inhibitors can increase sildenafil exposure two- to three-fold. However, co-administration with bosentan, a CYP3A4 inducer, can significantly reduce sildenafil concentrations. This is important because about 20%-40% of children with PH eventually receive dual therapy with a PDE-5 inhibitor and endothelin receptor antagonist, usually sildenafil and bosentan (71, 72). Emerging data from genome-wide association studies have identified variants in the *PFAS* and *CAMK2D* genes that affect treprostinil dose requirements by influencing cyclic AMP production (73).

There are currently no published clinical practice guidelines recommending the use of pharmacogenetics to guide medication selection or dosing in the medical treatment of PH. While genetic testing is recommended for diagnostic and prognostic purposes, as well as family counseling in PH, existing guidelines emphasize that genetic test results are not meant to guide treatment (74, 75). Further, there are no clinical trials specifically investigating pharmacogenetic-guided therapy selection or dosing in pulmonary hypertension are described in the available literature.

## Conclusion

Implementation of pharmacogenetics in pediatric cardiology shows promise for optimizing cardiovascular drug dosing, improving patient care and preventing adverse events. The current landscape of gene-specific, evidence-based dosing recommendations provide actionable frameworks for tailoring therapy to individual patients. International regulations generally permit genetic testing of minors when it offers direct clinical benefit, while predictive testing for adult-onset disorders is often restricted or discouraged to preserve the child's future autonomy. Common objections include concerns regarding informed consent, psychological harm, privacy, discrimination, and incidental findings. In the context of pharmacogenetic testing, when results have immediate and future implications for medication safety and efficacy, the benefits may outweigh the concerns (76, 77). Additionally, significant barriers remain to routine implementation (78, 79).

Barriers to consistent implementation include limited provider education and/or familiarity, variability in access to rapid and cost-effective genetic testing, and challenges in integrating results into clinical workflows. Although CPIC guidelines provide strong recommendations for select gene-drug pairs, evidence gaps remain, particularly for pediatric patients and those with congenital or structural heart disease. Currently, available evidence remains limited to predominantly adult populations. Developmental changes in drug metabolism represent a unique aspect to drug metabolism in pediatric patients. This complicates extrapolation from adult data but represents a unique opportunity for further research. These limitations underscore the need for continued investigation, including prospective clinical trials and real-world implementation studies to better define the clinical utility and cost-effectiveness of pharmacogenetically-guided therapy. Experience from routine clinical practice and implementation programs will be critical for generating practical evidence to inform and refine pharmacogenetically-guided therapy.

Moving forward, the incorporation of pharmacogenetics into pediatric cardiology practice will depend not only on expanding the evidence base but also on developing scalable models for clinical integration. This may include embedding decision support tools into electronic health records as an adjunct. As precision medicine initiatives continue to evolve, pharmacogenetics is poised to play a central role in personalizing clinical care in pediatric cardiology.

## Data Availability

The original contributions presented in the study are included in the article/Supplementary Material, further inquiries can be directed to the corresponding author.
